# COVID-19 incidence, severity, medication use, and vaccination among dentists: survey during the second wave in Brazil^*^

**DOI:** 10.1590/1678-7757-2022-0016

**Published:** 2022-10-03

**Authors:** Rafael R MORAES, Marcos B CORREA, Paulo R MARTINS-FILHO, Giana S LIMA, Flavio F DEMARCO

**Affiliations:** 1 Universidade Federal de Pelotas Brasil Universidade Federal de Pelotas, Brasil.; 2 Universidade Federal de Sergipe Brasil Universidade Federal de Sergipe, Brasil.

**Keywords:** COVID-19 vaccines, Vitamin D, Zinc, Ivermectin, Chloroquine

## Abstract

**Objective:**

This cross-sectional study with dentists in Brazil assessed the COVID-19 incidence and severity, its vaccination status, and the level of confidence in vaccines in May 2021 (COVID-19 second wave). The medications used to prevent or treat COVID-19, including controversial substances (vitamin D, ivermectin, zinc, and chloroquine), were analyzed.

**Methodology:**

Dentists were recruited by email and responded to a pretested questionnaire until May 31, 2021. Bivariate and multivariate regression analyses were performed (α=0.05). Prevalence ratios were calculated for the association between professional characteristics and two outcomes: SARS-CoV-2 infection and use of controversial substances.

**Results:**

In total, 1,907 responses were received (return rate of 21.2%). One third of dentists reported intermediate levels of confidence in the safety and efficacy of COVID-19 vaccines, but 96% had received at least one vaccine dose, mainly CoronaVac. The effect of the pandemic on dental practice was classified as lower/much lower, in comparison with the first wave, by 46% of participants. Moreover, 27% of dentists had already tested positive for SARS-CoV-2 and about 50% had relatives or friends who had been hospitalized or died from COVID-19. At least one medication was used by 59% of participants and 43% used two or more substances. Vitamin D (41%), ivermectin (35%), and zinc (29%) were the most frequent substances. More experienced dentists (≥21 years of professional experience) were 42% more likely to use controversial substances than less experienced dentists. The prevalence of use of controversial substances was 30% higher among dentists with residency or advanced training, such as postgraduate degrees, in comparison with participants holding MSc or PhD degrees. Participants with low confidence in vaccines were 2.1 times more likely to use controversial substances than participants with a very high confidence.

**Conclusion:**

The results of this study show the high severity of the COVID-19 pandemic in Brazil and raised questions about the use of scientific evidence by dentists in their decision to use controversial substances.

## Introduction

The COVID-19 pandemic imposed significant challenges to dentistry worldwide.^1^ Dental practice during the pandemic was associated with negative feelings among dentists, who presented high anxiety levels and fear of exposure to SARS-CoV-2 and infection at work.^3^ A study during the first wave in Brazil showed that regional COVID-19 incidence and mortality rates were associated with fear of SARS-CoV-2 infection in dental offices.^3^ Since the beginning of the pandemic, the Brazilian government has been criticized for being hostile to scientific evidence and unable to stop the spread of SARS-CoV-2.^6,7^ Until June 2022, COVID-19 caused more than 666,000 deaths in Brazil, which is one of the highest global mortality rates (about 313 deaths per 100,000 inhabitants).^8^

Amid an epidemiological crisis, a topic that has received great attention worldwide is the use of ineffective or controversial substances to prevent or treat COVID-19.^9-12^ This issue was the subject of investigational hearings in the Brazilian Senate, including the off-label use of hydroxychloroquine and ivermectin, among others. A recent article showed that a small set of poorly designed studies on medications played a significant role in misinformation during the COVID-19 first wave in Brazil,^13^ when vaccines were still not available. In this turbulent scenario of uncertainty about the future of the pandemic combined with fear and high risk of exposure to SARS-CoV-2 in dental offices, dentists could be inclined to self-medicate or use substances without proven efficacy against COVID-19. At the same time, hesitation about accepting vaccination has been an issue worldwide^14,15^ and the level of confidence in COVID-19 vaccines could be associated with the use of unproved medications. A recent study performed in Italy showed that 18% of participating dentists were hesitant about COVID-19 vaccines^16^ whereas another study showed that the willingness to receive a COVID-19 vaccine was higher in South America than in the USA and Russia.^15^

This study aimed to assess the COVID-19 incidence and severity among dentists in Brazil, as well as the use of medications to prevent or treat COVID-19, information on vaccination status, and their level of confidence in vaccines. This study was carried out in May 2021, during the COVID-19 second wave, and about 7.9% of the Brazilian population had a confirmed COVID-19 diagnosis.^8^ The second wave was one of the most severe periods of the pandemic in Brazil, as more than 180,000 deaths from COVID-19 occurred from March to May 2021.^8^ The results of this study could help in understanding the severity of the pandemic among dentists, as well as help dental and other health professionals to better understand the effect of COVID-19 and strengthen preparedness for future infectious disease outbreaks.

## Methodology

### Study design and ethical aspects

This cross-sectional study was performed in Brazil in May 2021, one year after the performance of a similar study with dentists in Brazil during the COVID-19 first wave.^3^ The study protocol was approved by the institutional review board (Protocol No. 4.015.536) and all research methods were in accordance with the Declaration of Helsinki. This study mainly aimed to address the effect of COVID-19 on dental practice and its associated aspects, including COVID-19 incidence, severity, medication use, and vaccination among dentists. A questionnaire which was developed and pretested in previous studies was used.^3,17^ All participants had to agree to participate in the study to access the questionnaire. They were instructed to print or save the first page of the questionnaire to retain a copy of the informed consent form. In accordance with open science practices, the study project, the questionnaire (in its original language), and the database of responses are available in an open platform (https://osf.io/dnbgs; DOI:10.17605/OSF.IO/DNBGS). An English translation of the questionnaire is available at https://dx.doi.org/10.17605/OSF.IO/DNBGS. The Consensus-Based Checklist for Reporting of Survey Studies (CROSS) was used.^18^

### Questionnaire development, content, and pretesting

Details on the development and pretesting of the original questionnaire were previously published.^3,17^ A self-administered electronic questionnaire was used. It underwent minor revisions, including the exclusion of 11 and the addition of five questions most of them about COVID-19 severity, medication use, and vaccination which were not present in the previous versions of the questionnaire. The new questions were formulated based on the inputs of three researchers in three discrete rounds of revision.

The questionnaire was created on SurveyMonkey (Momentive Inc., San Mateo, CA, USA). Its first section presented the title and objective of the study and informed that the invitation was extended only to dentists. This section provided the informed consent form, which explained that the participation was voluntary and unpaid and showed the potential risks and benefits of the study. Moreover, it assured that all responses would be anonymous and confidential. Multiple participations from a same respondent were not allowed by the surveying system, which also protected the questionnaire from unauthorized access. Each question was presented to participants only after they responded to the previous question, showing that there were no discrete screens. The questionnaire had 25 mandatory items (one open- and 24 close-ended questions) about demographic and professional characteristics (n=6), professional practices and challenges during the pandemic (n=13), and COVID-19 vaccination, prevalence, severity, and medication (n=6). The items were not randomized and no adaptive questioning methods were used. All responses could be revised using back buttons at any time before submitting the completed form. In order to reduce bias related to response errors (units), the options “I’d rather not say,” “I don’t know how to answer it,” and “Does not apply to me” were available in all close-ended questions.

### Sample selection, participant recruitment, and survey administration

A total of 24,392 registered dentists were invited to participate by an email sent via SurveyMonkey (convenience sampling). The list was provided by the Brazilian Ministry of Health in 2020 and did not include all dentists registered in Brazil, but professionals from all Brazilian states working in public and/or private dental networks. This list was used as it was received—no selection was carried out. It also included dentists who participated in the first study in May 2020.^3^ In previous studies, these sources promoted sample variability and corresponded to the general population of registered dentists in Brazil.^3,17^ The email was a brief invitation with the study objective, the average response time (5 min), and the notification of the university conducting the study. The questionnaire was tested for its possibility to be well read on different computers, tablets, and mobile phones. The first emails were sent on May 13, 2021. Reminder emails were sent seven and 11 days after to reduce non-response bias. Considering a number of about 350,000 dentists in Brazil, 1,530 responses would be necessary to ensure a 95% confidence interval and 2.5% margin of error. Responses were collected until May 31, 2021.

### Data analysis

To reduce non-response error, the partial completion of questionnaires was not allowed (completion proportion=100%). In some questions, responses were restricted to a specific population—only dentists assisting patients when the study was performed, for example. The responses “I’d rather not say,” “I don’t know how to answer it,” and “Does not apply to me” were considered missing data. No strategies for weighting items, propensity scores, or sensitivity analysis were used. In analyses using data from the question on medication use, vitamin D, ivermectin, zinc, and chloroquine/hydroxychloroquine were considered controversial substances. Descriptive statistics were used to identify variable frequencies and distributions with respective 95% confidence intervals (CI). Bivariate and multivariate Poisson regression analyses were performed and prevalence ratios (PR) were estimate for the association between professional characteristics and two COVID-19-related outcomes: SARS-CoV-2 infection and use of controversial substances by dentists. Variable selection in the multivariate models was performed using the backward stepwise method. Variables from the bivariate analysis were considered only if p<0.25. All analyses were performed in Stata 14.2 (StataCorp, College Station, TX), considering α=0.05.

## Results

Of the 24,392 emails sent to dentists, 1,347 bounced (loss of 5.5%) and 9,010 were opened (unique visitors), as registered by the surveying system (view proportion=36.9%). A return rate of 21.2% was calculated from the opened emails and 1,907 valid responses were received from all 26 Brazilian states and the Federal District.

### Sample characteristics

The length of work experience and levels of postgraduate education varied among participants (Table 1). There was a predominance of responses from women and dentists in the public sector. In total, 88.7% of participants worked in Southern, Southeastern, and Northeastern Brazil. By May 2021, 96% of dentists had received at least one dose of the COVID-19 vaccine and the most common vaccine was CoronaVac (65%). There was a predominance of high and very high levels of confidence in COVID-19 vaccines (58%), but one third of the sample reported intermediate levels of confidence in the safety and efficacy of the vaccines.

**Table 1:** Characteristics of the sample of Brazilian dentists, 2021 (n=1,907)

Variable/category	n*	%	95% CI
**Sex**	**1,907**		
Women	1,414	74.1	72.1; 76.1
Men	493	25.9	23.9; 27.9
**Years of professional experience**	**1,905**		
≤5	451	23.7	21.8; 25.6
6–10	474	24.9	23.0; 26.9
11–20	534	28.0	26.1; 30.1
>20	446	23.4	21.6; 25.4
**Postgraduate education (complete)**	**1,899**		
None	505	26.6	24.7; 28.7
Residency or advanced special training	997	52.5	50.2; 54.7
MSc or PhD	397	20.9	19.1; 22.8
**Main work sector**	**1,892**		
Public	1,106	58.5	56.1; 60.6
Private	613	32.4	30.3; 34.5
Other	173	9.1	8.0; 10.7
**Region of Brazil**	**1,906**		
South	586	30.7	28.7; 32.9
Southeast	552	29.0	26.8; 30.9
Northeast	552	29.0	27.1; 31.2
Central West	139	4.0	3.2; 5.0
North	77	7.3	6.2; 8.6
**COVID-19 vaccination status**	**1,894**		
Not vaccinated	76	4.0	3.2; 5.0
Partially vaccinated	188	9.9	8.5; 11.2
Fully vaccinated	1,63	86.1	84.6; 87.7
**Vaccines received**	**1,818**		
CoronaVac (Sinovac/Butantan)	1,179	64.9	62.7; 67.1
Oxford (AstraZeneca/Fiocruz)	622	34.2	32.0; 36.4
Pfizer (BioNTech)	17	0.9	0.5; 1.4
**Level of confidence in the safety and efficacy of COVID-19 vaccines**	**1,856**		
Very low	59	3.2	2.3; 4.0
Low	104	5.6	4.1; 6.3
Intermediate	611	32.9	30.5; 35.2
High	712	38.4	36.3; 41.1
Very high	370	19.9	18.4; 22.4
**Effect of the COVID-19 pandemic on dental practice in comparison with one year before (May 2020, first wave in Brazil)**	**1,749**		
Much lower	154	8.8	7.5; 10.4
Lower	648	37.1	36.0; 40.8
Similar	528	30.2	26.5; 31.0
Higher	240	13.7	12.5; 15.9
Much higher	179	10.2	8.6; 11.6
**How prepared do you feel to treat patients with COVID-19?**	**1,743**		
Not at all prepared	412	23.6	21.7; 25.9
Poorly prepared	268	15.4	14.1; 17.7
Moderately prepared	486	27.9	25.5; 29.9
Well prepared	434	24.9	22.6; 26.9
Very well prepared	143	8.2	6.9; 9.7
**Fear of being infected at work**	**1,766**		
None	354	20.0	18.0; 22.0
Little	553	31.3	29.5; 34.2
Moderate	450	25.5	23.6; 28.0
High	409	23.2	20.5; 24.7
**Frequency of use of N95 masks in dental appointments**	**1,71**		
Never	100	5.9	4.8; 7.1
Perceived higher risk of COVID-19	64	3.7	2.9; 4.8
Aerosol-generating procedures	127	7.4	6.3; 9.0
Whenever it is available	237	13.9	11.7; 15.1
Always	1,182	69.1	67.2; 71.8
**Frequency of use of face shields in dental appointments**	**1,699**		
Never	159	9.4	7.6; 10.5
Perceived higher risk of COVID-19	90	5.3	4.5; 6.8
Aerosol-generating procedures	438	25.8	23.8; 28.2
Always	1,012	59.6	57.1; 62.0
**Have you ever treated patients with confirmed COVID-19?**	**1,752**		
No or does not know	1,279	73.0	70.9; 75.1
Yes	473	27.0	24.9; 29.1
**Tested positive for SARS-CoV-2**	**1,754**		
No	1,288	73.4	71.2; 75.4
Yes	466	26.6	24.6; 28.8
**COVID-19 severity**	463		
Asymptomatic	55	11.9	9.1; 15.2
Mild	352	76.0	71.9; 79.8
Severe	48	10.4	7.7; 13.5
Need for hospitalization	8	1.8	0.7; 3.4
**Severe COVID-19 among relatives or friends**	**1,717**		
None	878	51.1	48.7; 53.5
Yes, and hospitalization	367	21.4	19.5; 23.4
Yes, and death	472	27.5	25.7;29.7
Substances used to prevent or treat COVID-19**	**1,554**		
None of the substances listed below	639	41.1	38.7; 43.6
Vitamin D	630	40.5	38.0; 43.0
Ivermectin	549	35.3	32.9; 37.8
Zinc	450	29.0	26.8; 31.4
Azithromycin***	416	26.8	24.6; 29.0
Corticosteroid	190	12.2	10.6; 14.0
Chloroquine or hydroxychloroquine	69	4.4	3.4; 5.5
Remdesivir***	2	0.1	0.0; 0.4
**Number of substances used to prevent or treat COVID-19**	**1,554**		
1	251	16.2	14.4; 18.1
2	252	16.2	14.4; 18.1
3	217	14.0	12.3; 15.9
4 or more	195	12.5	10.9; 14.2
**Most frequent combinations of substances to prevent or treat COVID-19**	**1,554**		
Vitamin D + zinc + ivermectin	106	6.8	5.6; 8.2
Vitamin D + zinc	101	6.5	5.3; 7.8
Chloroquine/hydroxychloroquine + ivermectin + zinc + vitamin D	61	3.9	3.0; 5.0
Ivermectin + zinc	53	3.4	2.6; 4.4

CI: confidence interval. *Varies from total n due to missing data in different questions. **More than one answer was possible. ***Prescription medication.

### Current effect of COVID-19 on dental practice

The effect of the pandemic on dental practice during the second wave was classified as lower/much lower, in comparison with the first wave, by 46% of participants. However, 39% felt poorly or not prepared to treat patients with COVID-19 and 49% reported moderate or high fear of being infected with the SARS-CoV-2 during work. N95 masks and face shields were always used in dental appointments by at least 60% of dentists and 27% treated patients with confirmed COVID-19.

### COVID-19 incidence, severity, and medication use

In total, 27% of dentists had already tested positive for SARS-CoV-2 by May 2021 and most of them were asymptomatic or had mild symptoms (88%). Moreover, 49.8% had relatives or friends who had been hospitalized or died from COVID-19. Regarding medication use, 58.9% of participants used at least one of the seven controversial substances listed in Table 1 to prevent or treat COVID-19. Vitamin D was the most common (41%), followed by ivermectin (35%), zinc (29%), and azithromycin (27%). The use of chloroquine or hydroxychloroquine was not prevalent (4%). A total of 74.7% of participants who tested positive for SARS-CoV-2 used at least one of these substances whereas the frequency of this use among dentists who tested negative was 52.4%. In total, 42.7% of dentists used two or more substances. The most frequent combination was vitamin D and zinc combined or not with ivermectin.

Table 2 presents the prevalence ratios for the association between professional characteristics and SARS-CoV-2 infection. Sex, work sector, and years of professional experience were not associated with the prevalence of infection. Dentists working in Northern Brazil were 58% more likely to be infected than those working in Southern Brazil. Dentists with residency or advanced training, such as postgraduate education, were 26% more likely to be infected than professionals with MSc or PhD degrees. The level of confidence in COVID-19 vaccines was not associated with a history of infection. Participants who reported no fear of being infected at work were 48% more likely to be infected with COVID-19 than those who reported high fear.

**Table 2:** Crude (c) and adjusted (a) prevalence ratios (PR) for the association between professional characteristics and COVID-19 infection. Multivariate Poisson regression analysis (n=1,907)

Variable	PR^**c**^	95% CI	PR^**a**^	95% CI
**Sex**				
Men	1		*	*
Women	0.97	0.81; 1.15		
**Main work sector**				
Public	1		*	*
Private	0.87	0.73; 1.74		
Others	0.84	0.62; 1.13		
**Years of professional experience**				
≤10	1		*	*
11–20	1.29	1.05; 1.59		
≥21	1.17	0.93; 1.48		
**Region of Brazil**				
South	1		1	
Southeast	0.77	0.62; 0.95	0.78	0.63; 0.98
Northeast	1.12	0.92; 1.35	1.16	0.95; 1.41
North	1.52	1.10; 2.11	1.58	1.14; 2.20
Central West	0.94	0.68; 1.31	0.94	0.67; 1.32
**Postgraduate education**				
MSc or PhD	1		1	
Residency or advanced special training	1.27	1.03; 1.57	1.26	1.02; 1.57
None	1.13	0.88; 1.44	1.13	0.88; 1.45
**Confidence in COVID-19 vaccines**				
Very high	1		*	*
High	1.18	0.94; 1.49		
Moderate	1.24	0.98; 1.57		
Low	1.33	0.92; 1.93		
Very low	1.60	1.07; 2.38		
**Fear of being infected at work**				
High	1		1	
Moderate	1.03	0.81; 1.31	1.04	0.82; 1.32
Little	0.97	0.77; 1.22	0.99	0.79; 1.25
None	1.43	1.14; 1.80	1.48	1.18; 1.85

CI: Confidence Interval. *Not included in the multivariate analysis.

Table 3 presents the prevalence ratios for the association between professional characteristics and the use of controversial substances in the multivariate analysis. Sex and work sector were not associated with the use of controversial substances. However, more experienced dentists (>21 years of professional experience) were 42% more likely to use controversial substances than less experienced dentists. Participants from the Central Western, Northern, and Northeastern Brazil were from 29% to 37% more likely to use controversial substances than participants from Southern Brazil. The prevalence of use of controversial substances was 30% higher among dentists with residency or advanced training, such as postgraduate education, in comparison with participants holding MSc or PhD degrees. The level of confidence in COVID-19 vaccines also influenced medication use. The increased prevalence of controversial medication use was associated with decreased levels of confidence in vaccines. Participants with low confidence in COVID-19 vaccines, for instance, were 2.1 times more likely to use controversial substances than participants with a very high confidence in vaccines.

**Table 3:** Crude (c) and adjusted (a) prevalence ratios (PR) for the association between professional characteristics and the use of controversial substances by Brazilian dentists. Multivariate Poisson regression analysis (n=1,907)

Variable	PR^**c**^	95% CI	PR^**a**^	95% CI
**Sex**				
Men	1		1	
Women	1.11	0.98; 1.25	1.09	0.97; 1.23
**Main work sector**				
Public	1		1	
Private	1.09	0.99; 1.22	1.10	0.99; 1.23
vOther	0.56	0.43; 0.74	0.76	0.58; 1.00
**Years in practice**				
≤10	1		1	
11–20	1.17	1.04; 1.33	1.18	1.03; 1.35
≥21	1.38	1.22; 1.55	1.42	1.26; 1.61
**Region of Brazil**				
South	1		1	
Southeast	0.99	0.86; 1.15	0.98	0.85; 1.13
Northeast	1.29	1.14; 1.48	1.37	1.20; 1.57
North	1.37	1.08; 1.73	1.37	1.07; 1.76
Central West	1.29	1.07; 1.57	1.29	1.07; 1.57
**Postgraduate education**				
MSc or PhD			1	
Residency or advanced special training	1.52	1.30; 1.77	1.30	1.11; 1.53
None	1.31	1.10; 1.57	1.19	0.98; 1.43
**Confidence in COVID-19 vaccines**				
Very high	1		1	
High	1.38	1.14; 1.66	1.31	1.09; 1.58
Moderate	1.90	1.59; 2.27	1.65	1.37; 1.99
Low	2.21	1.77; 2.76	1.94	1.55; 2.42
Very Low	2.50	1.97; 3.16	2.14	1.67; 2.75

CI: Confidence Interval.

## Discussion

This study showed a high prevalence of COVID-19 among Brazilian dental professionals (27%) and a frequent occurrence of hospitalization and death from this disease among their relatives or friends during the second wave. Moreover, 59% of participants used one or more substances to prevent or treat COVID-19, including vitamin D, zinc, and ivermectin, which have limited evidence to support their clinical use against COVID-19.

Studies conducted in several countries showed varying prevalence of COVID-19 among dentists: 1.1% in Brazil (May 2020),^3^ 9.1% in Belgium (July–Sept. 2020),^19^ 2.6% in the USA (June–Nov. 2020),^20^ 4.9% in Latin America (Sep.–Dec. 2020),^21^ 10.9% in Italy (Dec. 2020–Jan. 2021),^16^ and 1.1% in Canada (July 2020–Feb. 2021).^22^ In a multi-country study performed in 2020, about 15% of dentists reported COVID-19 symptoms^23^and a prevalence rate of 25% was observed in Czech Republic (June 2021).^24^ The differences in contraction rates could be partially explained by distinct public health measures in response to the pandemic and different COVID-19 spreading rates in the countries. In this study, for example, the prevalence of COVID-19 was higher in Northern than in Southern Brazil, which could be related to the abrupt increase in the number of cases in Manaus, Northern Brazil, during the first months of 2021.^25,26^ The high prevalence of dentists who tested positive for COVID-19 could also be associated with the long-lasting trend of new daily cases of this disease in Brazil,^8^ which continued to have high rates of virus transmission during 2020 (first wave) and had an increase in number of cases in the first half of 2021 (second wave).

About 50% of participants had relatives or friends who had been hospitalized or died from COVID-19. This result is worrying and highlights the severity of the pandemic in Brazil, as COVID-19 was the leading cause of deaths in the public health system during 2020 and 2021.^27^ Although the effect on dental practice in May 2021 seemed to be lower when compared with May 2020, participants still frequently felt poorly prepared to treat patients with COVID-19 and had moderate to high fear of being infected by this disease at work. Dentists who reported no fear of being infected by the disease were more likely to be infected by COVID-19, which raises questions about the influence of the more or less strict preventive measures adopted by them. Moreover, the high risk of SARS-CoV-2 infection in dental offices, along with the frequent occurrence of COVID-19 among dentists’ relatives and friends, could be associated with high levels of psychosis and anxiety,^2,5^ which may help to explain the high prevalence of use of off-label medication for COVID-19.

Misinformation has been a major problem in the pandemic and a potential source of public confusion and controversy. A study in Vietnam showed that more than 91% of health professionals learned about COVID-19 via social media,^28^ which are digital places where the content of information is not policed.^29^ Health professionals are expected to keep themselves up to date with reliable information to educate and treat their patients. However, they have been reported as the major practitioners of self-medication.^30,31^ Scientific literature plays an important role in this context of professional practices. This is highlighted by the observation that dentists holding MSc or PhD degrees were less likely to use controversial substances. This finding could be related to the type of education and the generally longer duration of MSc and PhD courses when compared with specialized clinical training courses. We also found that more experienced dentists were more likely to use controversial substances than less experienced dentists, which could be associated with the higher risk of older adults of having severe cases of COVID-19. Participants with low confidence in COVID-19 vaccines were 2.1 times more likely to use controversial substances than participants with a high confidence in these vaccines. To the best of our knowledge, this is the first study to show this association.

Among many factors that may negatively interfere with evidence-based health practice in the pandemic context, there is the large number of low-quality studies reporting conflicting results for the treatment of COVID-19. This is associated with difficulties of implementing the best available evidence due to lack of time, knowledge, or skills to critically evaluate the literature. This means that controversial substances could be considered either effective or ineffective depending on the article selected and how it was interpreted. Regarding vitamin D, for instance, a systematic review suggested that its supplementation was associated with reduced intensive care unit (ICU) admission, the need for mechanical ventilation, and mortality.^32^ Another systematic review showed that vitamin D did not reduce the risk of these clinical outcomes.^33^ A meta-analysis showed that zinc reduced COVID-19 death rates^34^ whereas a different meta-analysis showed that there is no evidence to support zinc supplementation in patients with COVID-19.^35^ Karale et al.^36^ (2021) stated that the treatment of COVID-19 with ivermectin may reduce the need for hospitalization, but a living network meta-analysis showed that it was highly uncertain whether ivermectin used as a preventive measure would reduce the risk of SARS-CoV-2 infection.^37^ Another frequent problem is the presence of methodological issues across the primary studies. In all aforementioned systematic reviews, the authors highlighted that more randomized controlled trials with larger sample sizes and less risk of bias, imprecision, and/or heterogeneity were necessary. It seems that the topic of controversial substances to prevent or treat COVID-19 will still attract attention in the following years, as definitive conclusions will hardly be accepted universally. In the meantime, dentists are encouraged to rely on evidence with low risk of bias and good methodological quality or evidence-based guidelines, when available.

A positive finding of this study was the high rate of COVID-19 vaccination. In Brazil, health professionals were priorities for vaccination, followed by older adults, thus, dentists were vaccinated in the first stages of the immunization program with the first vaccines available in Brazil. This explains the high frequency of dentists who received CoronaVac, which was the first vaccine authorized for emergency use by the Brazilian Health Regulatory Agency in January 2021. This vaccine was developed by the *Instituto Butantan* in association with the Chinese laboratory Sinovac. A study showed that the rapid increase in vaccination of Brazilian older adults with CoronaVac was associated with a significant decline in mortality.^38^ This vaccine was criticized by President Bolsonaro and his allies during its testing phases in 2020. In that period, a study showed that Brazilians were less likely to accept vaccination when the country of origin of the vaccine was mentioned.^39^ Since January 2022, four vaccines have been available for use in Brazil and dentists throughout the country are eligible to take booster shots. Further studies could evaluate the acceptance of dental professionals to the new phases of COVID-19 vaccination and the maintenance of other preventive measures to address whether the so-called “pandemic fatigue” may decrease their adherence to individual and collective risk reduction strategies.^40^

This study had limitations and care should be taken when extrapolating its results. Participants were free either to accept or not the invitation to participate in the study, which may have led to self-selection bias, increasing the chances of dentists who were more concerned about the pandemic and perhaps more willing to use medications to participate. Moreover, we did not collect data on doses or frequency of use of substances, which may have varied greatly among participants. Self-reported SARS-CoV-2 infection was another limitation, as the diagnosis could be influenced by variations in quality and accuracy across molecular and serologic tests. Moreover, self-reported medication use may have been influenced by social-desirability bias, but the questionnaire was anonymous and thus this influence could be low. A strength of this study was that a large sample of dentists was recruited in a period when Brazil was struggling to deal with the pandemic. Further studies could address the use of controversial substances and the socioeconomical aspects involved.

## Conclusion

This study with dentists during the COVID-19 second wave in Brazil showed a high incidence of this disease among dentists, a frequent occurrence of hospitalization and death from COVID-19 among their relatives or friends, and a very frequent use of controversial substances to prevent or treat COVID-19. The COVID-19 vaccination status was high among the studied dentists. The overall findings highlighted the high severity of the pandemic in Brazil and raised questions about the use of scientific evidence by dentists in their decision to use controversial substances, such as vitamin D, zinc, and ivermectin.

## References

[1] Novaes TF, Jordão MC, Bonacina CF, Veronezi AO, Araujo CA, Olegário IC (2021). COVID-19 pandemic impact on dentists in Latin America’s epicenter: São-Paulo, Brazil. PLos One.

[2] Mekhemar M, Attia S, Dörfer C, Conrad J (2021). The psychological impact of the COVID-19 pandemic on dentists in Germany. J Clin Med.

[3] Moraes RR, Correa MB, Queiroz AB, Daneris A, Lopes JP, Pereira-Cenci T (2020). COVID-19 challenges to dentistry in the new pandemic epicenter: Brazil. PLos One.

[4] Sharma A, Chhabra KG, Bhandari SS, Poddar G, Dany SS, Chhabra C (2021). Emotional well-being of dentists and the effect of lockdown during the COVID-19 pandemic: a nationwide study. J Educ Health Promot.

[5] Chen Y, Li W (2021). Influencing factors associated with mental health outcomes among dental medical staff in emergency exposed to Coronavirus Disease 2019: a multicenter cross-sectional study in China. Front Psychiatry.

[6] Ortega F, Orsini M (2020). Governing COVID-19 without government in Brazil: ignorance, neoliberal authoritarianism, and the collapse of public health leadership. Glob Public Health.

[7] Boschiero MN, Palamim CV, Ortega MM, Mauch RM, Marson FA (2021). One year of Coronavirus Disease 2019 (COVID-19) in Brazil: a political and social overview. Ann Glob Health.

[8] World Health Organization (c2022). WHO Coronavirus (COVID-19) dashboard.

[9] Lasco G, Yu VG (2021). Pharmaceutical messianism and the COVID-19 pandemic. Soc Sci Med.

[10] Arshad AR, Ijaz F, Siddiqui MS, Khalid S, Fatima A, Aftab RK (2021). COVID-19 pandemic and antimicrobial resistance in developing countries. Discoveries (Craiova).

[11] Duncan EM, Goulao B, Clarkson J, Young L, Ramsay CR (2021). “You had to do something”: prescribing antibiotics in Scotland during the COVID-19 pandemic restrictions and remobilisation. Br Dent J.

[12] Martins PR, Ferreira LC, Heimfarth L, Araújo AA, Quintans LJ (2021). Efficacy and safety of hydroxychloroquine as pre-and post-exposure prophylaxis and treatment of COVID-19: a systematic review and meta-analysis of blinded, placebo-controlled, randomized clinical trials. Lancet Reg Health Am.

[13] Alves CP, Barreto Segundo JD, Costa GG, Pereira-Cenci T, Lima KC, Demarco FF (2021). How a few poorly designed COVID-19 studies may have contributed to misinformation in Brazil: the case for evidence-based communication of science. BMJ Open Sci.

[14] Dror AA, Eisenbach N, Taiber S, Morozov NG, Mizrachi M, Zigron A (2020). Vaccine hesitancy: the next challenge in the fight against COVID-19. Eur J Epidemiol.

[15] Solís Arce JS, Warren SS, Meriggi NF, Scacco A, McMurry N, Voors M (2021). COVID-19 vaccine acceptance and hesitancy in low- and middle-income countries. Nat Med.

[16] Belingheri M, Roncalli M, Riva MA, Paladino ME, Teruzzi CM (2021). COVID-19 vaccine hesitancy and reasons for or against adherence among dentists. J Am Dent Assoc.

[17] Moraes RR, Correa MB, Daneris A, Queiroz AB, Lopes JP, Lima GS (2021). Email vs. Instagram recruitment strategies for online survey research. Braz Dent J.

[18] Sharma A, Duc NT, Thang TL, Nam NH, Ng SJ, Abbas KS (2021). A consensus-based checklist for reporting of survey studies (CROSS). J Gen Intern Med.

[19] Carvalho JC, Declerck D, Jacquet W, Bottenberg P (2021). Dentist related factors associated with implementation of COVID-19 protective measures: a national survey. Int J Environ Res Public Health.

[20] Araujo MW, Estrich CG, Mikkelsen M, Morrissey R, Harrison B, Geisinger ML (2021). COVID-2019 among dentists in the United States: a 6-month longitudinal report of accumulative prevalence and incidence. J Am Dent Assoc.

[21] Moraes R, Cuevas-Suárez C, Escalante-Otárola W, Fernández M, Dávila-Sánchez A, Grau-Grullon P (2021). A multi-country survey on the impact of COVID-19 on dentists in Latin America. Res Square.

[22] Madathil S, Siqueira WL, Marin LM, Sanaulla FB, Faraj N, Quiñonez CR (2022). The incidence of COVID-19 among dentists practicing in the community in Canada: a prospective cohort study over a six-month period. J Am Dent Assoc.

[23] COVIDental Collaboration Group (2021). The COVID-19 pandemic and its global effects on dental practice. An International survey. J Dent.

[24] Schmidt J, Perina V, Treglerova J, Pilbauerova N, Suchanek J, Smucler R (2021). COVID-19 Prevalence among Czech Dentists. Int J Environ Res Public Health.

[25] Ferrante L, Steinmetz WA, Almeida ACL, Leão J, Vassão RC, Tupinambás U (2020). Brazil’s policies condemn Amazonia to a second wave of COVID-19. Nat Med.

[26] Sabino EC, Buss LF, Carvalho MP, Prete CA, Crispim MA, Fraiji NA (2021). Resurgence of COVID-19 in Manaus, Brazil, despite high seroprevalence. Lancet.

[27] Castro MC, Gurzenda S, Turra CM, Kim S, Andrasfay T, Goldman N (2021). Reduction in life expectancy in Brazil after COVID-19. Nat Med.

[28] Huynh G, Nguyen TH, Tran V, Vo K, Vo V, Pham L (2020). Knowledge and attitude toward COVID-19 among healthcare workers at District 2 Hospital, Ho Chi Minh City. Asian Pac J Trop Med.

[29] Apuke OD, Omar B (2021). Fake news and COVID-19: modelling the predictors of fake news sharing among social media users. Telemat Inform.

[30] Sisay M, Mengistu G, Edessa D (2018). Epidemiology of self-medication in Ethiopia: a systematic review and meta-analysis of observational studies. BMC Pharmacol Toxicol.

[31] Sadio AJ, Gbeasor-Komlanvi FA, Konu RY, Bakoubayi AW, Tchankoni MK, Bitty-Anderson AM (2021). Assessment of self-medication practices in the context of the COVID-19 outbreak in Togo. BMC Public Health.

[32] Hariyanto TI, Intan D, Hananto JE, Harapan H, Kurniawan A (2022). Vitamin D supplementation and Covid-19 outcomes: a systematic review, meta-analysis and meta-regression. Rev Med Virol.

[33] Rawat D, Roy A, Maitra S, Shankar V, Khanna P, Baidya DK (2021). Vitamin D supplementation and COVID-19 treatment: A systematic review and meta-analysis. Diabetes Metab Syndr.

[34] Beran AB, Mhanna M, Srour O, Ayesh H, Abdulsattar W, Khokher W (2021). Effect of micronutrient supplements on COVID-19: a systematic review and meta-analysis. Chest.

[35] Szarpak L, Pruc M, Gasecka A, Jaguszewski MJ, Michalski T, Peacock FW (2021). Should we supplement zinc in COVID-19 patients? Evidence from meta-analysis. Pol Arch Intern Med.

[36] Karale S, Bansal V, Makadia J, Tayyeb M, Khan H, Ghanta SS (2021). Clinical outcomes associated with ivermectin use in covid-19: an updated systematic review and meta-analysis. Chest.

[37] Bartoszko JJ, Siemieniuk RA, Kum E, Qasim A, Zeraatkar D, Ge L (2021). Prophylaxis against COVID-19: living systematic review and network meta-analysis. BMJ.

[38] Victora CG, Castro MC, Gurzenda S, Medeiros AC, França GV, Barros AJ (2021). Estimating the early impact of vaccination against COVID-19 on deaths among elderly people in Brazil: analyses of routinely-collected data on vaccine coverage and mortality. EClinicalMedicine.

[39] Gramacho WG, Turgeon M (2021). When politics collides with public health: COVID-19 vaccine country of origin and vaccination acceptance in Brazil. Vaccine.

[40] Trogen B, Caplan A (2021). Risk compensation and COVID-19 vaccines. Ann Intern Med.

